# The Exposure Status of Environmental Chemicals in South Korea: The Korean National Environmental Health Survey 2018–2020

**DOI:** 10.3390/toxics12110829

**Published:** 2024-11-19

**Authors:** Sooyeon Hong, Ok-Jin Kim, Sun Kyoung Jung, Hye Li Jeon, Suejin Kim, Jihyon Kil

**Affiliations:** Environmental Health Research Department, National Institute of Environmental Research, Ministry of Environment, Incheon 22689, Republic of Koreasuenier@korea.kr (S.K.)

**Keywords:** Korean National Environmental Health Survey (KoNEHS), environmental chemicals, human biomonitoring

## Abstract

In South Korea, a Human Biomonitoring (HBM) program, known as the Korean National Environmental Health Survey (KoNEHS), was launched in 2009. This study aims to provide an overview of environmental chemical exposures in South Korea based on data from the KoNEHS cycle 4 (2018–2020). To ensure population representativeness, Koreans aged 3 years and older were recruited from 426 sites across the country. A total of 6381 participants joined in the collection of biospecimens, which were subsequently analyzed for 33 environmental chemicals or their metabolites, including nine that were not included in the previous cycle. The five most common PFASs were detected in more than 99.7% of the participants. The GM of serum PFOS was the highest in adults at 15.1 µg/L (13.9, 16.4) and in adolescents at 7.97 µg/L (7.42, 8.56). In adults, there was a gradual decrease in the detection rate and concentration of some heavy metals and phthalate metabolites. In children and adolescents, the detection rate of BPA in urine decreased, while the rate of its substitutes BPF and BPS increased, and the rate of propyl paraben in urine decreased significantly. The results of the KoNEHS cycle 4 indicate that exposure levels to certain environmental chemicals are still high, highlighting further monitoring and on-going surveys to determine their trends, especially for newly investigated substances, such as PFASs.

## 1. Introduction

According to UNEP’s Global Chemicals Outlook, global chemical sales are projected to grow from €3.47 trillion in 2017 to €6.6 trillion by 2030 [1]. In addition, in 2018, the European Environment Agency reported that approximately 62% of chemicals consumed in the EU in 2016 were hazardous to health [2], yet the risks and exposure levels of most chemicals remain unknown [3]. Exposure to chemicals is becoming increasingly complex due to changes in the production, trade, and use of chemicals; the prevalence of chemical mixtures; and growing awareness of chemicals among the public and policymakers [1]. Human Biomonitoring (HBM) is a reliable method for assessing human exposure to chemicals from different sources and by different pathways [4,5,6]. It is one of the most important parts of the environmental health surveillance system, especially to measure the effects of environmental chemicals on health and to help prevent diseases by assessing the extent of exposure and health risks [7]. The determination of chemicals in human blood and urine was first used in the field of occupational medicine. Lead [8] or benzene metabolites [9] in human body fluids are early examples of HBM workplace exposures [6]. The main purpose of national environmental health policy is to analyze the causes of and establish comprehensive management plans for health effects caused by various sources of environmental pollution [10]. Therefore, identifying the level of human exposure to environmental pollutants and major exposure routes is an essential step for establishing environmental health management plans. In 2005, Korea started a national human biomonitoring as a pilot project called “Study on the concentration of heavy metals in the Korean blood” [7]. The results of the study were used to investigate the concentration of environmental chemicals in the human body, providing a legal basis for enacting the Environmental Health Act in 2008. The aim of this study is to introduce the KoNEHS cycle 4 (2018–2020) and provide an overview of the current status of exposure to environmental chemicals in South Korea.

## 2. Methods

### 2.1. The Korean National Environmental Health Survey (KoNEHS)

KoNEHS is an ongoing Korean representative population-based biomonitoring program in South Korea. It was launched by the National Institute of Environmental Research (NIER), Ministry of Environment (ME) in 2009, in accordance with Article 14 of the Environmental Health Act, monitoring the environmental chemical exposure and its health impacts every 3 years (Table A1). The details of KoNEHS have been previously reported [11,12,13]. The age range of the target population has been expanded from adults over 18 years to all population groups aged 3 years and older since the cycle 3 (2015–2017).

A total of 6381 Koreans aged 3 years and older participated in the KoNEHS cycle 4 (2018–2020) at 426 sites nationwide. The study measured 33 environmental chemicals or their metabolites in participants’ blood and urine, including nine newly added chemicals (five per- and polyfluoroalkyl substances, two phthalate metabolites, butyl paraben, and benzophenone-3) compared to the previous cycle study. Data were also collected on demographic and socioeconomic characteristics, lifestyle, dietary habits, and personal care products use through age-specific questionnaires.

### 2.2. Sampling Design

#### 2.2.1. Adults Survey

The target population for the adult survey was Koreans aged 19 years and older who live in South Korea. The general framework of the sampling design and sampling methodology for the adult survey was the same as the previous KoNEHS cycles [11,12]. The sampling frame used in the sampling design for the household survey was the census tract list of the 2015 Population and Housing Census [14]. To ensure national population representativeness and to better reflect the distribution of characteristics of environmental chemicals concentrations in 2015, KoNEHS used a two-stage stratified sampling method. The primary stratification criterion was based on regions, including five administrative provinces, metropolitan cities and municipalities, and coastal regions reflecting topographical characteristics. Air pollution monitoring sites were also included to investigate the correlation between air pollution levels and health effects. The second stratification criterion considered socioeconomic factors such as housing types and residential area characteristics, classifying a total of 69 strata. The adult sample size for the cycle 4 was 3675 adults with 15 from each of the 245 survey sites, taking into accounts the efficiency of the cycle 3, margin of errors, and budget. The number of enumeration sites allocated to each stratum was calculated using the square root proportional distribution method for the total number of households in the stratum. Applying this method was deemed to be advantageous for regional estimation and to increase the sampling rate in provinces with relatively few households, which could increase the statistical accuracy of the estimates [13].

#### 2.2.2. Preschoolers, Children, and Adolescents Survey

The target populations of KoNEHS cycle 4 of preschoolers, children, and adolescents included domestic residents aged 3 to 18 years, and the sampling design and methodology were the same as those used in the previous KoNEHS cycle 3 [13]. The sampling frame was based on the most recent list of childcare and education institutions available at the time of the survey [14,15,16,17]. The survey population was considered representative of the target population—number of subjects/age groups × 100% was more than 92% for preschoolers and 97% for children and adolescents [18]. The same stratified sampling method applied to adults was used for preschoolers, children, and adolescents. Nine strata were selected based on five administrative provinces and municipalities, with a second stratification for the early childhood and school surveys based on the types of childcare providers and schools. The sample size for the KoNEHS cycle 4 of preschoolers, children, and adolescents was determined by considering issues such as efficiency, margin of errors, and budget from the KoNEHS cycle 3.

The sample size was 504 preschoolers from 56 daycare centers and kindergartens (an average of 9 per institution), 704 schoolers from 69 elementary schools (an average of 12 per school), and 804 schoolers from 67 middle and high schools (an average of 12 per school) for the surveys of preschoolers, children and adolescents, respectively. Similarly to the adult survey, the number of samples allocated to each stratum was based on a square root proportional distribution using the square root of the number of students in each stratum. This ensured that areas with relatively small sample sizes secured a larger number of samples to enhance statistical reliability. The sample of young children was drawn by probability proportional to the number of schools or nurseries in each stratum [19].

### 2.3. Participants Recruitment and Fieldwork

#### 2.3.1. Participants Recruitment

Participants for the adult survey were recruited through on-site visits to the sampled sites. As a first step, 2015 Population and Housing Census dwelling maps [14] were used to identify tract boundaries. This approach aimed to ensure that no households were missed, and to identify any gaps in the characteristics of the survey sites, such as changes in administrative areas or redevelopment. The next step was the verification of the actual residency of the people recorded in the Census, creating a KoNEHS household register for eligible households, and selecting the sample households using a cluster sampling method based on the household register. During the on-site visits to the sampled households, adults aged 19 years and older were invited to participate in the survey with the information on the KoNEHS provided. The final step was to obtain consent forms from those who agreed to participate in the survey and collect information to be entered in the household register. In collaboration with the Ministry of Education and the Ministry of Health and Welfare, preschoolers, children, and adolescents were recruited through close contact with the administrators of the sampled institutions: daycare centers, kindergartens, and schools. Representatives from the sampled organizations were provided with information on the study including the number of participants needed from their organizations. Additionally, parents of eligible participants received information on the study through home communications and were encouraged to participate. The list of eligible participants was finalized by checking the list submitted by agency representatives on the KoNEHS system.

#### 2.3.2. Fieldwork

The fieldwork consisted of physical measurements, questionnaires, and biospecimen collection (blood and urine), with details varying by age group. For adults and adolescents, anthropometric measurements included height, weight, and waist circumference, while height and weight were measured for other age groups. Questionnaires were used to assess demographic and socioeconomic variables, as well as diet, housing, lifestyle, and health behaviors that were potential sources of exposure to environmental toxicants. For biospecimens, both urine and blood samples were collected from adults and adolescents, while only urine samples were collected from other age groups. In general, fieldwork involved the following steps: identifying a personal ID, obtaining consent for the survey, conducting physical measurements, surveying and reviewing via 1:1 interview, and collecting biospecimens. For adults, this entire process took place at the local survey site. Preschooler and child surveys required caregivers to complete a questionnaire and blood samples were not collected. Questionnaires and urine sample collection tools were distributed to households prior to the fieldwork. and survey staff visited institutions or schools to collect completed questionnaires and urine samples on the day of the fieldwork. The adolescent survey included blood sampling in collaboration with a hospital near schools. Selected adolescents visited the hospital to provide blood and urine samples prior to the fieldwork. On the day of the fieldwork, a surveyor visited schools to conduct physical measurements and surveys, and the samples from the hospital were collected by a specialized specimen transport organization. However, due to the COVID-19 pandemic that began in late 2019, blood was not collected from adults who participated in the 2020 survey. Similarly to the children’s survey, questionnaires and urine collection tools were delivered before the survey and collected on the day of the fieldwork.

Samples collected through KoNEHS were managed and processed in accordance with the Standard Methods for the Management of Biospecimens in Basic Research [20,21] (CDC, 2018; NIER, 2022c). This approach aimed to minimize possible errors in the sequence of biospecimen collection, management, and transport, ensuring the accuracy and reliability of the analytical results. A brief sample processing methodology is outlined below. Blood samples were collected in 21 mL blood collection vacuum tubes to obtain whole blood and serum samples, while urine samples were collected in 60 mL tubes by random sampling of midstream urine. Whole blood samples collected during the fieldwork were placed in a rolling mixer for 30 min to ensure good mixing with anticoagulants, while serum was held for 30 min and centrifuged. Urine samples were immediately immersed in ice water after collection to prevent the temperature change, and then transported at low temperature (2–8 °C). Temperature changes during transportation were monitored in real time. Samples were centrally aliquoted within 24 h of collection and stored frozen at −70 °C until analysis.

### 2.4. Laboratory Analysis

PFOA, PFOS, PFHxS, PFNA and PFDeA are among the persistent pollutants investigated for the first time in the KoNEHS cycle 4. Additionally, two relatively low molecular weight metabolites of phthalates, DEP (diethyl phthalate) and DMP (dimethyl phthalate), as well as butyl paraben and benzophenone-3, were newly analyzed. The five types of PFASs were analyzed using a Q-Sight Triple Quad high-performance liquid chromatograph/mass spectrometer (Perkin Elmer, Waltham, MA, USA). The limit of detection (LOD) for PFASs in serum according to this test method were as follows: PFOA was 0.050 μg/L, PFOS was 0.056 μg/L, PFHxS was 0.071 μg/L, PFNA was 0.019 μg/L, and PFDeA was 0.017 μg/L. Phthalate metabolites were analyzed by Agilent 1200 series/Agilent 6490 HPLC. The detection limit of phthalate metabolites in urine was MMP at 0.069 μg/L and MEP at 0.108 μg/L. Parabens were measured by API triple Quad 5500 LC-MS/MS (AB Sciex, Framingham, MA, USA). The detection limit of butylparaben and benzophenone-3 in urine was 0.109 μg/L and 0.129 μg/L, respectively.

The analyses of environmental chemicals and urinary creatinine in KoNEHS were performed by specialized laboratories validated by proficiency assessment using certified standard reference materials—SRM, Standard Reference Material; CRM, Certificate Reference Material; G-EQUAS, German External Quality Assessment Scheme. The analytical laboratories performed the analyses according to the manual for laboratory procedures on the KoNEHS cycle 4 [22,23], and the analytical equipment for each chemical is listed in the Appendix (Table A2). The analytical laboratory calculated the limit of detection (LOD) and the intermediate detection limit (IDL) when the analytical conditions changed or every six months. They determined the low, medium, and high control of the QC samples, verifying the accuracy by the degree control value within the control standard (mean ± 2SD), and determining the precision by repeating the measurement within and between lots. If a technical error in the pretreatment process was recognized, or if the measured value of the QC sample was outside the acceptable range or exceeded the calibration line, the sample was reanalyzed according to the established procedure [13].

### 2.5. Statistical Analysis

Representative values for environmental chemicals from the KoNEHS cycle 4 by weighting methods. Weights were calculated by combining design weights, adjustments for non-response, and adjustments for post-stratification based on each age group included in the sampling design. The weighting approach ensured that the sample structure closely mirrored the population structure for each sex and age group, thereby improving the accuracy of the estimates. If the concentration of environmental chemicals was below the limit of detection (LOD), it was replaced by the LOD/√2 value [24]. Environmental chemicals in urine were adjusted for creatinine. Given that the KoNEHS cycle 4 collected random urine samples, the concentration of urine and the amount of excretion may affect the concentration values of environmental chemicals [25,26,27]. Additionally, creatinine concentrations outside the range of 0.3 to 3.0 g/L were excluded from all analyses [28]. When reporting the geometric mean and percentiles of creatinine-adjusted concentrations, we applied the same LODs as for the concentrations using weight per volume of urine (unadjusted results) [24].

The arithmetic mean, geometric mean and 95% confidence interval of the geometric mean, and percentiles (P25, P50, P75, P90, P95) were calculated to determine the distribution of the concentrations of environmental chemicals in the bio-samples. The geometric mean was considered because the concentrations of environmental chemicals tend to be skewed to one side of the distribution. The detection rates were defined as the proportion (%) of assay results that are above the LOD. For variables known to influence concentrations of environmental chemicals, such as sex, residence, and smoking status, the survey calculated the geometric mean and 95% confidence interval of the geometric mean for each group, and presented the *p*-value for the difference in means. The geometric mean and 95% confidence interval were compared by age and survey periods to identify minor trends in the concentrations of environmental chemicals that were monitored continuously since the previous cycle.

Statistical analyses of all these environmental chemical concentration values were performed using the survey procedure in SAS 9.4 (SAS Institute, Cary, NC, USA), which reflects a multistage sampling design.

### 2.6. Ethical Considerations

All KoNEHS surveys were conducted with the informed consent of participants or their legal representatives. Consent for the collection of personal information, the collection and use human bodily fluids, and the participation in research was obtained from adults aged 19 years and older. For those under 12 years of age, consent was obtained from a legal representative, such as a parent. For those aged 12 to 18 years consent was obtained from a parent or legal representative to participate in the survey. KoNEHS cycle 4 was approved by the Research Ethics Committee of the NIER, Ministry of the Environment (IRB No. NIER-2018-BR-003-02, 2018).

## 3. Results

### 3.1. Survey Participants

The characteristics of participants in KoNEHS cycle 4 are shown in Table 1. The number of participants in KoNEHS cycle 4 was 6381, of which 4239 were aged 19 years and older, 828 were aged 12–18, 736 were aged 6–11, and 578 were aged 3–5. By sex, adults were slightly more likely to be female (50.2%), while adolescents, children, and preschoolers were slightly more likely to be male (51.2–51.8%). Across all age groups, there was a high proportion of participants living in urban areas, with over 74.5% living in such areas. The Body Mass Index (BMI) showed that the proportion of obese people increased with age, with the highest proportion (44.5–50.3%) in the group earning between $2350 and $5875 per month. Regarding smoking history, 63.3% of adults and 93.2% of adolescents reported never having smoked, while 44.7% of parents of children and 40.4% of parents of preschoolers reported being current smokers. More than 50% of all age groups reported using a drinking water filter, and more than 53% of all age groups reported using coated kitchen utensils at least once a week. When asked about the frequency of shellfish consumption, the highest proportion of all age groups reported eating shellfish at least once a month (41.3–48.0%).

### 3.2. The Status of Exposure to Environmental Chemicals

The results of KoNEHS, which are recognized as national statistics, are published on the Korean statistical information service [29] by Statistics Korea. The concentrations of the main substances for heavy metals are as follows: the geometric means (GM) of urinary cadmium concentrations were 0.349 μg/L (95% confidence interval (CI) = 0.313, 0.389), [0.394 μg/g crea. (0.351, 0.441)] for adults, 0.146 μg/L (0.127, 0.167), [0.077 μg/g crea. (0.067, 0.088)] for adolescents, and 0.197 μg/L (0.0183, 0.214), [0.177 μg/g crea. (0.159, 0.184)] for children. In preschoolers, the geometric mean could not be calculated with more than 40% of the measurements below the LOD. These values are significantly lower than the concentration limits set by the German Human Biomonitoring (HBM) commission’s recommended “no risk of adverse health effects level” HBM-I value [30] (adults—1 μg/L, children and adolescents—0.5 μg/L, 2011). The geometric mean of blood mercury concentrations was 2.96 μg/L (2.82, 3.10) in adults and 1.38 μg/L (1.33, 1.44) in children and adolescents, which are also well below the concentration standards set by the German HBM- I value [30] (children and adults—5 μg/L, 1999). In phthalates, the urinary concentrations of two diethylhexyl phthalate (DEHP) metabolites (MEHHP+MEOHP), which are used as plasticizers, were 32.2 μg/L (29.4, 35.2), [39.1 μg/g crea. (36.3, 42.1) in preschoolers and children, 39.3 μg/L (35.7, 43.3), [35.7 μg/g crea. (33.6, 38.1)] middle and high school students 19.1 μg/L (15.1, 24.2), [10.7 μg/g crea. (8.31, 13.7)] and adults 16.8 μg/L (15.7, 17.9), [20.2 μg/g crea. (19.1, 21.3)] and the concentrations of phthalate metabolites in all age groups were well within the concentration standards set by the German HBM-I values [30] (women of childbearing age; 200 μg/L, general population 500 μg/L, 2007). The analysis results for the newly introduced environmental chemicals in KoNEHS cycle 4 are shown in Table 2.

Five perfluorinated compounds in serum showed detection rates greater than 99.7% in both adults and adolescents. The geometric mean serum concentration of PFOA, the most common perfluorinated chemical, was 6.43 μg/L (6.16, 6.71) in adults and 3.66 μg/L (3.44, 3.89) in adolescents, while the geometric mean serum concentration of PFOS was 15.1 μg/L (13.9, 16.4) in adults and 7.97 μg/L (7.42, 8.56) in adolescents. Although these values are lower than the German Human Biomonitoring Commission’s recommended “health concern level” HBM-II [31] (General population—PFOA 10 μg/L, PFOS 20 μg/L, 2020), they are more than three times higher than those described in Germany’s HBM-I values [32] (General population—PFOA 2 μg/L, PFOS 5 μg/L, 2020) for both adolescents and adults, and two to seven times higher than those described in the United States, Canada, and France for both adolescents and adults (Table 2).

In addition, among the newly analyzed urinary phthalate metabolites, the detection rate of MMP was more than 95%, and the detection rate of MEP was more than 87%, except for the population aged 3 to 5 years (detection rate of 63.7%). The geometric mean of MMP concentrations in urine ranged from 2.27 to 5.05 μg/g crea. with the highest concentration in preschoolers and a decreasing trend with increasing age. On the other hand, MEP concentrations ranged from 1.11 to 5.87 μg/g crea. with the lowest concentrations in preschoolers. Compared to international concentrations, MMP concentrations were slightly higher, but MEP concentrations were significantly lower. The detection rate of butyl paraben was more than 91%, and the detection rate of benzophenone-3 was more than 80%. The geometric mean of butyl paraben concentrations in urine ranged from 0.666 to 1.16 μg/g crea. and benzophenone-3 concentrations ranged from 0.676 to 0.820 μg/g crea. with the highest concentrations in adults. Compared to measurements in other countries, butyl paraben was found to be high, but benzophenone-3 was found to be significantly lower. To examine differences in concentrations according to potential sources and routes of exposure, perfluorinated compound concentrations were compared by sex, region of residence, BMI, monthly income, smoking status, food consumption, and coated cookware. In the adult population, region of residence, BMI, smoking history, food consumption, coated cookware, and frequency of shellfish consumption were broadly associated with perfluorinated compound concentrations (*p* < 0.05, 0.001). In contrast, in the adolescent population, the use of coated cookware, and frequency of shellfish consumption were associated (*p* < 0.05, *p* < 0.001) (Table 3).

### 3.3. Temporal Trends in Environmental Chemical Concentrations in KoNEHS

**Figure 1:**
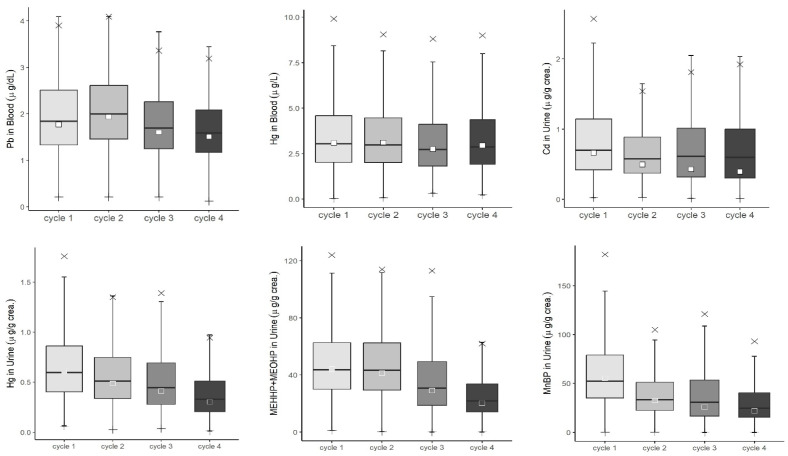
Levels of environmental chemicals measured in the population aged 19 years and old in Korean National Environment Health Survey 2009–2020 (weighted results). NOTE: ⅹ indicates p95, transparent square box indicates geometric mean, and lower bar means minimum value.

Among the environmental chemicals monitored by KoNEHS, we examined minor changes in substances measured over two or more cycles. In adults, the trend of the concentration values of the analyzed environmental chemicals from cycle 1 to 4 was confirmed by the results for subjects aged 19 years and older (Figure 1). For heavy metals, blood lead concentrations (µg/dL) were 1.77 in cycle 1, 1.94 in cycle 2, 1.60 in cycle 3, and 1.51 in cycle 4. Blood mercury concentrations (µg/L) were 3.08, 3.11, 2.75, and 2.96, with detection rates of 98–99%, showing similar levels from cycle 1 to 4. However, urinary mercury concentrations (µg/g crea.) decreased from 0.611 to 0.487, 0.412, and 0.303, with detection rates of 92–98%, and urinary cadmium concentrations (µg/g crea.) decreased slightly from 0.664, 0.496, 0.429, and 0.394, with detection rates of 99.5%, 98.0%, 93.2%, and 92.4%. The concentration values of MEHHP+MEOHP (µg/g crea.), a metabolite of DEHP, decreased to 44.1, 41.2, 29.0, and 20.2, but detection rates were above 98.8% from cycle 1 to 4. The concentration values of MnBP (µg/g crea.) decreased to 55.2, 32.4, 26.0, and 22.0 over time, with detection rates slightly decreasing to 99.3%, 97.5%, 98.2%, and 96.7%.

**Figure 2:**
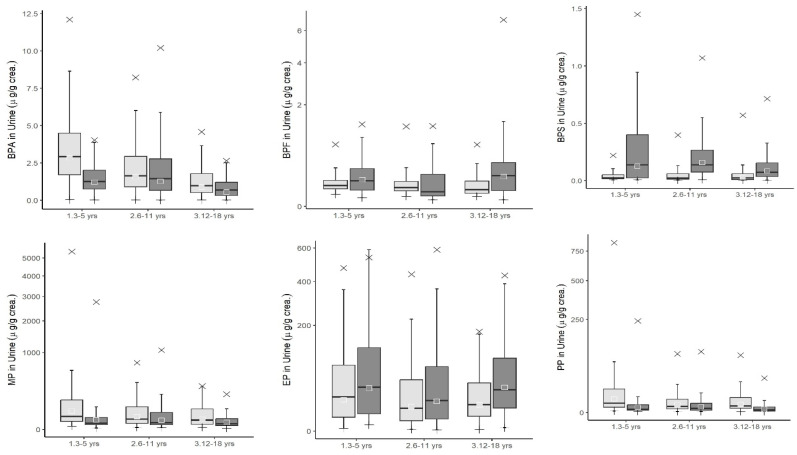
Levels of environmental chemicals measured in the population aged from 3 to 18 years in Korean National Environment Health Survey 2009–2020 (weighted results). NOTE: ⅹ indicates p95, transparent square box indicates Geometric Mean, and lower bar means minimum value. Light gray bar means KoNEHS cycle 3, and dark gray bar means KoNEHS cycle 4.

Preschoolers, children, and adolescents were included in the study from KoNEHS cycle 3 onwards and we compared the results between the two cycles. Concentrations of endocrine disruptors were determined for children aged 3 to18 years (Figure 2). The concentrations of bisphenol A (µg/g crea.) decreased over time from 2.83 in preschoolers, 1.56 in children, and 0.886 in adolescents in cycle 3 to 1.20, 1.26, and 0.554 in cycle 4. In contrast, bisphenol F (µg/g crea.), an alternative to bisphenol A, increased in concentration to 0.137 in preschoolers and 0.172 in adults in cycle 4 from below the LOD in cycle 3, while remaining below the LOD in children in cycle 4. Bisphenol S (µg/g crea.), another alternative to bisphenol A, increased in concentration from below the LOD in preschoolers and children in cycle 3 and 0.033 in adolescents to 0.126 in preschoolers, 0.158 in children, and 0.086 in adolescents in the cycle 4.

**Figure 3:**
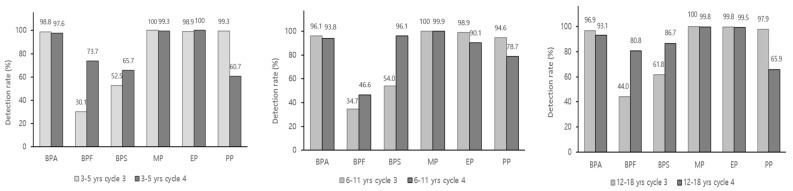
Detection rate of environmental chemicals measured in the population aged 3 to 18 years in Korean National Environment Health Survey 2009–2020 (weighted results).

The study found that the detection rates of bisphenol A alternatives, bisphenol F and bisphenol S, varied significantly (Figure 3). The detection rate of bisphenol F increased from 30.1% to 73.7% in preschoolers 34.7% to 46.6% in children, and 44.0% to 80.8% in adolescents, while the detection rate of bisphenol S increased from 52.5% to 65.7% in preschoolers, from 54.0% to 96.1% in children and 61.8% to 86.7% in adolescents.

The concentration values (µg/g crea.) of methyl and propyl parabens decreased from 56.4 and 5.41 in preschoolers, 26.5 and 1.70 in children, 16.7 and 1.97 in adolescents in cycle 3, to 15.1 and 0.761 in preschoolers, 13.9 and 0.938 in children, 7.87 and 0.374 in adolescents in cycle 4. However, the concentration values (µg/g crea.) of ethyl paraben increased by 1.5 to 2.6-fold from 17.4, 10.6 and 12.2 to 32.9, 15.8 and 34.0 in each group, respectively. The detection rate of methyl and ethyl paraben did not change significantly between the cycle 3 and the cycle 4, but the detection rate of propyl paraben showed a significant decrease from 99.3 to 60.7% in preschoolers, from 94.6 to 78.7% in children and from 97.9 to 65.9 in adolescents.

## 4. Discussion

### 4.1. Status of Exposure to Environmental Chemicals

The KoNEHS cycle 4 (2018–2020) study is a national survey to investigate the expo-sure levels of the Korean population based on a multistage sampling of 6381 people. We analyzed the KoNEHS data on the exposure of the Korean population to environmental chemicals and compared it with data published in other countries.

The serum concentrations of per- and polyfluoroalkyl substances (PFASs) newly added in KoNEHS cycle 4 were similar to or slightly higher than those in the previous Korean studies [33,34,35]. Compared to national biomonitoring concentrations in the United States, Canada, France, and Germany [36,37,38,39], they were significantly higher. Internationally, PFOS was listed as a target substance under the Stockholm Convention in 2009, followed by PFOA in 2019 and PFHxS and its salts in 2022 [40]. As a result, North America and Europe have implemented PFASs regulations earlier than Asia [41,42]. In comparison, China has implemented restrictions and bans on the production and use of PFOA and PFOS since 2024 [43]. In Korea, the Persistent Organic Pollutants Control Act, enacted in 2008, has regulated and administered PFOA since 2019 and PFOS since 2009, However, there is an exception of PFOA used in semiconductor coatings, which is classified as a permanent exemption until alternative technologies are secured, and a specific exemption for plating agents [40]. Due to their persistence in the environment, we can be exposed to PFASs in trace amounts through food and environmental media and can remain in the body for long periods of time [44,45]. In addition, the volatile precursors of PFASs can travel long distances through the atmosphere [46,47]. These properties have led to their widespread detection in the general population without occupational exposure [48]. Various routes of exposure to PFASs in humans have been suggested, including diet, drinking water, food packaging and non-stick cookware, household dust, and outdoor and indoor air [33,49,50,51,52]. The analysis of KoNEHS for individual exposures was consistent with a study [53] which found that individuals who frequently used coated (non-stick) utensils showed had higher levels than those who did not.

Drinking water is a major source of exposure to PFASs worldwide. It has been reported that PFASs emissions from PFASs production facilities to the atmosphere and human consumption can enter the soil and groundwater and contribute to drinking water sources [54]. In Korea, the water quality monitoring standard for perfluorinated compounds in drinking water has been set at 0.07 ug/L for PFOA, 0.48 ug/L for PFOS and 0.07 ug/L for PFHxS since 2019 [55]. In addition, to prevent perfluorinated chemicals from migrating into food, the government is tightening regulations on exposure, including bans on the use of perfluorinated chemicals in the manufacture of utensils, containers, and packaging from 2020 [56]. Additionally, diet is considered one of the major exposure pathways for PFASs [49,57,58]. The contribution of specific food types to PFAS concentrations varies by geographical region, population characteristics, and between specific PFAS compounds [33,58,59]. Shellfish consumption was positively correlated with PFAS concentrations in Korea [35].

MMP, a metabolite of DMP, was found to be at similar or slightly lower levels than in other countries, while MEP, a metabolite of DEP, was found to be at very low concentrations compared to the United States, Canada, Germany, and France [38]. Low molecular weight phthalates, such as DMP and DEP, are mainly added to household chemicals, such as fragranced cosmetics and personal care products [60]. In particular, DEP has been detected in fragranced products (e.g., perfumes) and artificial nails [61]. According to the national chemical distribution data [62], the use of DMP and DEP is increasing every year, but there are no regulations in Korea. KoNEHS will continue to analyze the concentration of MMP and MEP, metabolites of DMP and DEP, in the body.

For environmental phenolics, butyl paraben and benzophenone-3 were added to cycle 4. For butyl paraben, concentrations in KoNEHS cycle 4 were lower than those found in Canada [63], but higher than those found in the United States and Germany [37,38,64]. Parabens have been reported to be more effective as antifungals when used in combination than alone [65] and are added in mixtures to food and personal care products (PCPs) [66,67]. In some countries, butyl paraben is regulated by restricting its formulation or setting maximum allowable concentrations, depending on the intended use. In Korea, butyl paraben is banned as a food additive, but its use in cosmetics, quasi-drugs, and hygiene products is allowed under safety standards for human exposure [53]. Compared to the United States and Canada, benzophenone-3 was found at very low levels in all age groups [37]. In Korea, benzophenone-3 is used in cosmetics and hair products at concentrations up to 5% due to its ability to penetrate the skin and protection from UV rays [68].

### 4.2. Temporal Trends in Environmental Chemical Concentration in KoNEHS

Monitoring temporal changes in the substances measured since 2009 and has found that heavy metal concentrations in South Korea have been gradually decreasing. In the case of blood lead levels, a declining trend over time has been observed in many countries [69,70,71]. In Korea, the decrease in atmospheric lead concentrations is consistent with study results indicating a reduction in lead concentrations in the body [72]. In China, a study investigating changes in lead concentrations from 2010 to 2018 found that lead concentrations decreased due to China’s lead reduction policies [73]. For mercury, previous studies have confirmed the association between smoking and mercury concentrations, indicating that fish and shellfish may have the greatest impact on dietary mercury exposure [74,75]. In Korea, mercury has only been used for purposes permitted under the Minamata Convention on Mercury since 2020, and its release into the air, soil, and water has been controlled [47]. Urinary mercury concentrations have shown a decline over time in South Korea, and the U.S. NHANES results also confirm that urinary mercury concentrations in adults are about 50% lower than those measured 10 years ago, indicating a decrease over time [76].

A study found that concentrations of MEHHP+MEOHP (µg/g crea.), a metabolite of DEHP, were slightly decreasing, indicating a correlation between the observed levels and the substitution of DEHP with other phthalates in industry [77]. Since 2021, Korea has restricted the use of phthalates in electrical and electronic products in compliance with the European Union’s Restriction of Hazardous Substance (RoHS), an international environmental standard and strengthened regulations on hazardous substances by expanding the range of equipment subject to the ban [78]. According to the Canadian DEHP Risk Management Evaluation Report, Canada has implemented risk management measures to reduce DEHP exposure, including adding DEHP to the Cosmetic Ingredient Watch List, implementing a phthalates regulation, and conducting continuous monitoring on hazardous chemicals in food and food packaging. Additionally, subsequent CHMS biomonitoring results have shown a gradual decrease in Canadian exposure to DEHP [79].

The findings of KoNEHS exhibited higher concentrations of the alternatives, BPF and BPS, than BPA in preschoolers, children, and adolescents. The use of BPA has been restricted in several countries [80], and the Korean Ministry of Food and Drug Safety amended relevant regulations in 2019 to include baby products, food containers, and packaging [53]. The findings of decreasing concentrations of BPA and increasing concentrations of BPF and BPS are consistent with the findings that BPF and BPS are widely used as substitutes for BPA [81]. However, BPF and BPS are structurally similar to BPA, which limits their effectiveness as less toxic alternatives [82,83]. More recently, animal studies have reported neurological disorders associated with BPF [84] highlighting the difficulty of substituting chemicals that are safe from environmental and human toxicity [85].

Cross-sectional studies cannot determine a causal relationship between the potential exposure sources studied and the measured contaminant concentrations, so the associations highlighted in the analysis should be interpreted with caution [86]. This is evidently relevant for biomarkers with short half-lives [87,88,89,90]. However, the large overall sample size of KoNEHS allows for population-based estimates. Furthermore, the KoNEHS data collected according to a standardized protocol ensures the validity of our results. The study had few limitations aside from low participation among adults aged 19 to 35 years due to the recruitment and fieldwork conducted after working hours to encourage participation. However, sampling design characteristics were considered in the statistical analysis were considered, including sampling design weights, adjustment for nonresponse, and adjustment for post-stratification, thereby indicating that our estimates are representative of the population. Therefore, our population exposure estimates can be considered representative of Koreans living in South Korea between 2018 and 2020 and, demonstrating the adequacy identifying concentration levels for monitoring environmental toxicants.

## 5. Conclusions

In the modern world, the use of chemicals may be an unavoidable choice for producing necessary items. While the concentration of environmental hazardous substances in these items do not pose immediate risks, but significant efforts are required to use substances that minimize health effects for the future. Human biomonitoring in the Korean National Environmental Health Survey is a national effort to reduce chemical-related health effects.

## Figures and Tables

**Table 1:** Characteristics of the 6381 participants of KoNEHS cycle 4 (2018–2020).

**Characteristics**	**N (Weighted %) or Median (Min~Max)**			
	**Adults** (≥Aged 19 Years)	**Adolescents** (Aged 12–18 Years)	**Children** (Aged 6–11 Years)	**Preschoolers** (Aged 3–5 Years)
N	4239	828	736	578
Sex				
Male	1889 (49.8)	385 (51.8)	348 (51.4)	291 (51.2)
Female	2350 (50.2)	443 (48.2)	388 (48.6)	287 (48.8)
Age (years)	47.5 (19.0–82.0)	14.5 (12.0–17.0)	8.5 (6.0–11.0)	4.0 (3.0–5.0)
Study area ^1^				
Urban	2854 (74.7)	662 (84.8)	562 (77.3)	438 (85.7)
Rural	805 (10.2)	166 (15.2)	174 (22.7)	140 (14.3)
Coastal areas	213 (14.6)	-	-	-
Area near air monitoring station	367 (0.47)	-	-	-
Body Mass Index (kg/m^2^)				
Underweight (<18.5)	96 (2.9)	110 (13.7)	426 (58.3)	505 (87.6)
Normal (18.5–24.9)	2203 (53.0)	545 (64.8)	260 (34.9)	70 (12.1)
Obese (≥25.0)	1940 (44.1)	173 (21.5)	50 (6.8)	3 (0.45)
Monthly household income (US $) ^2^				
Do not know	36 (1.2)	47 (5.9)	26 (3.3)	15 (2.7)
<2350	1223 (22.6)	54 (6.5)	40 (5.4)	25 (4.0)
2350 to 5875	1845 (46.7)	377 (44.5)	358 (50.3)	264 (44.8)
5875 to 8225	682 (18.0)	203 (25.6)	206 (27.5)	166 (28.8)
>8225	453 (11.6)	147 (17.5)	106 (13.4)	108 (19.7)
Smoking status ^3^				
Non-smoker	2231 (63.3)	778 (93.2)	209 (27.6)	194 (32.3)
Former smoker	817 (18.0)	24 (3.3)	208 (24.7)	150 (27.3)
Current smoker	691 (18.7)	26 (3.5)	319 (44.7)	234 (40.4)
Drinking water				
Tap water	1055 (22.2)	174 (21.4)	139 (18.4)	97 (16.8)
Use a water purifier	2032 (50.7)	499 (61.3)	486 (66.5)	387 (67.3)
Bottled water	973 (24.0)	144 (16.4)	106 (14.4)	89 (15.3)
Mineral spring water	179 (3.2)	11 (1.0)	5 (0.7)	5 (0.6)
Use of nonstick kitchen tools				
No use	116 (2.4)	17 (2.2)	18 (2.3)	26 (4.0)
Less than once a week	232 (3.9)	35 (4.2)	25 (3.4)	13 (2.1)
Once a week and more	2328 (53.6)	482 (58.0)	420 (56.8)	308 (53.3)
Once a day and more	1563 (40.0)	294 (35.5)	273 (37.5)	231 (40.6)
Consumption of Shellfish				
Not eating	1627 (50.5)	281 (34.4)	304 (41.7)	232 (39.7)
Once a month and more	1128 (41.3)	435 (53.0)	340 (46.0)	270 (48.0)
Once a week and more	240 (8.2)	112 (12.6)	92 (12.3)	76 (12.3)

NOTE: Proportion of variables weighted. ^1^ Weighted N (%), Adults: Urban 32,813,160 (74.7), Rural 4,462,544 (10.2), Coastal areas 6,423,784 (14.6), Area near air monitoring station 19,168 (0.47), Adolescents: Urban 2,365,153 (84.8), Rural 425,229 (15.2), Children: Urban 2,120,391 (77.3), Rural 623,132 (22.7), Preschoolers: Urban 1,097,119 (85.7), Rural 183,666 (14.3), ^2^ Currency: 1175 won/US dollar, ^3^ Below 12 years, father or mother’s smoke habits.

**Table 2:** Levels of environmental chemicals newly added in the KoNEHS cycle 4 (2018–2020).

Environmental Chemical	Age Group (Years)	N	% > LOD ^1^	GM	(95% CI)	CHMS ^2^	Esteban ^3^	GerES ^4^	NHANES ^5^
Polyfluoroalkyl Substances (µg/L)									
PFOA	12–18	825	100	3.66	(3.44, 3.89)	0.96	1.56	0.994	1.18
	≥19	2993	100	6.43	(6.16, 6.71)	1.0	2.08	-	1.45
PFOS	12–18	825	100	7.97	(7.42, 8.56)	1.6	2.22	2.602	2.68
	≥19	2993	100	15.1	(13.9, 16.4)	2.3	4.03	-	4.50
PFHxS	12–18	825	99.7	2.52	(2.17, 2.92)	0.53	0.79	0.365	0.866
	≥19	2993	99.8	4.17	(3.88, 4.49)	0.70	1.37	-	1.11
PFNA	12–18	825	100	0.921	(0.867, 0.978)	0.34	0.61	<LOQ	0.348
	≥19	2993	100	2.06	(1.96, 2.17)	0.37	0.80	-	0.419
PFDeA	12–18	825	100	0.447	(0.426, 0.469)	NC	0.24	<LOQ	0.153
	≥19	2993	100	0.907	(0.864, 0.953)	0.11	0.34	-	0.199
Phthalate Metabolites (µg/g creatinine)									
MMP	3–5	541	97.6	5.05	(4.65, 5.48)	6.2	-	8.3	-
	6–11	721	100	3.40	(3.14, 3.68)	4.0	5.2	6.8	-
	12–18	736	96.4	2.27	(1.97, 2.61)	2.2		3.6	-
	≥19	3796	95.2	3.14	(2.89, 3.41)	1.7	3.6	-	-
MEP	3–5	541	63.7	1.11	(0.899, 1.37)	19.0	-	25.3	38.4
	6–11	721	100	5.87	(5.25, 6.56)	19.0	51.2	20.4	24.7
	12–18	736	90.5	4.26	(3.49, 5.19)	16.0		23.1	24.2
	≥19	3796	87.2	4.28	(3.62, 5.05)	16.0	71.4	-	28.9
Environmental Phenols (µg/g creatinine)									
Butyl paraben	3–5	541	100	0.666	(0.625, 0.711)	NC	-	NC	NC
	6–11	721	91.6	0.483	(0.414, 0.564)	NC	NC	NC	NC
	12–18	737	98.9	0.474	(0.444, 0.506)	NC		NC	NC
	≥19	3796	99.3	1.16	(1.09, 1.22)	NC	NC	-	NC
Benzophenone-3 (Oxybenzone)	3–5	541	88.1	0.676	(0.578, 0.791)	44.0	-	2.306	39.7
	6–11	721	85.9	0.790	(0.669, 0.934)	32.0	-	1.721	25.8
	12–18	737	89.6	0.537	(0.462, 0.624)	16.0	-	1.888	12.7
	≥19	3796	80.0	0.820	(0.746, 0.902)	17.0	-	-	20.1

NOTE: N, Sample size, LOD, Limit of detection, PFOA, Perfluorooctanoic acid, PFOS, Perfluoro octane sulfonic acid, PFHxS, Perfluoro hexane sulfonic acid, PFNA, Perfluoro nonanoic acid, PFDeA, Perfluoro decanoic acid, MMP, Monomethyl phthalate, MEP, Monoethyl phthalate, NC, geometric mean was not calculated, Missing: Polyfluoroalkyl Substances 12–18 (3), ≥19 (1246), Phthalate Metabolites 3–5(37), 6–11(15), 12–18 (92), ≥19 (443), Environmental Phenols 3–5(37), 6–11(15), 12–18 (91), ≥19 (443). ^1^ LOD (µg/L): PFOA 0.050, PFOS 0.056, PFHxS 0.071, PFNA 0.019, PFDeA 0.017, MMP 0.069, MEP 0.108, Butyl paraben 0.109, Benzophenone-3 0.129, ^2^ CHMS: Canadian Health Measures Survey (2018–2019): 12–19 years, 20–39 years, ^3^ Esteban: French ESTEBAN study (2014–2016): 6–17 years, 18–74 years, ^4^ GerES: German Environmental Survey (2014–2017): 3–5 years, 6–10 years, 14–17 years, ^5^ NHANES: US National Health and Nutrition Examination Survey (2017–2018): 3–5 years, 6–11 years, 12–18 years, Environmental Phenols (2015–2016).

**Table 3:** Serum geometric mean of polyfluoroalkyl substances (µg/L) in KoNEHS cycle 4 (2018–2020) participants.

	≥19 Years									
	N	PFOA			PFOS			PFHxS		
		GM	(95% CI)		GM	(95% CI)		GM	(95% CI)	
Sex										
Male	1298	6.73	(6.38, 7.11)	**	16.0	(14.6, 17.6)	**	4.77	(4.41, 5.16)	**
Female	1695	6.14	(5.83, 6.47)		14.2	(13.0, 15.4)		3.65	(3.36, 3.97)	
Study area										
Urban	2143	6.05	(5.79, 6.33)	**	13.7	(13.0, 14.4)	**	4.19	(3.91, 4.49)	**
Rural	447	6.32	(5.48, 7.28)		17.5	(15.2, 20.1)		3.92	(3.39, 4.54)	
Costal	133	9.11	(8.49, 9.77)		23.3	(15.4, 35.3)		4.20	(2.93, 6.03)	
Air monitoring area	270	6.10	(5.32, 6.99)		13.3	(11.6, 15.2)		4.42	(3.03, 6.45)	
Body Mass Index										
Underweight	52	4.16	(3.43, 5.03)	**	9.13	(6.83,12.2)	**	2.87	(2.26,3.64)	*
Normal	1489	6.22	(5.92, 6.54)		14.5	(13.3,15.7)		3.94	(3.59,4.30)	
Obese	1452	6.78	(6.43,7.15)		16.1	(14.5,17.8)		4.50	(4.19,4.84)	
Monthly household income (US $)										
Do not know	3	13.4	(13.2, 13.6)	**	33.8	(33.7, 34)	**	7.24	(7.14, 7.34)	**
<2350	873	7.63	(7.06, 8.24)		20.5	(17.3, 24.2)		5.09	(4.55, 5.70)	
2350 to 5875	1280	6.31	(6.04, 6.59)		14.3	(13.1, 15.7)		4.26	(3.98, 4.56)	
5875 to 8225	512	5.71	(5.26, 6.20)		13.2	(12.3, 14.2)		3.43	(3.09, 3.80)	
>8225	325	6.04	(5.34, 6.83)		12.8	(11.4, 14.3)		3.65	(3.05, 4.36)	
Smoking habits										
Never smoker	1930	6.07	(5.76, 6.40)	*	14.3	(13.1, 15.6)	**	3.71	(3.45, 3.98)	*
Former smoker	597	7.59	(7.02, 8.20)		18.5	(16.5, 20.7)		5.36	(4.82, 5.97)	
Current smoker	466	6.63	(6.18, 7.12)		14.8	(13.3, 16.6)		4.84	(4.24, 5.52)	
Drinking water										
Tap water	825	6.93	(6.45, 7.46)	*	17.9	(15.1, 21.2)	**	4.63	(4.17, 5.15)	*
Use a water purifier	1414	6.07	(5.78, 6.38)		14.0	(13.0, 15.0)		3.86	(3.57, 4.18)	
Bottled water	622	6.43	(5.82, 7.10)		14.0	(12.8, 15.3)		4.27	(3.73, 4.89)	
Mineral spring water	132	8.65	(7.06, 10.6)		20.0	(17.3, 23.2)		5.19	(4.34, 6.20)	
Use of nonstick kitchen tools										
No use	83	5.29	(4.10, 6.83)	*	13.8	(11.1, 17.1)	**	3.21	(2.54, 4.05)	*
Less than once a week	116	6.33	(5.50, 7.27)		15.1	(12.5, 18.4)		4.27	(3.52, 5.19)	
Once a week and more	1621	6.70	(6.38, 7.04)		15.6	(14.6, 16.6)		4.35	(4.05, 4.68)	
Once a day and more	1173	6.19	(5.64, 6.80)		14.6	(12.7, 16.7)		4.01	(3.50, 4.60)	
Consumption of Shellfish										
Not eating	1625	6.18	(5.87, 6.50)	**	15.3	(14.1, 16.6)	**	4.24	(3.95, 1.56)	**
Once a month and more	1128	6.47	(6.11, 6.86)		14.5	(13.2, 15.9)		3.94	(3.54, 4.39)	
Once a week and more	240	7.91	(6.95, 9.01)		16.5	(13.9, 19.5)		4.94	(3.96, 6.18)	
	**12–18 years**									
	**N**	**PFOA**			**PFOS**			**PFHxS**		
		**GM**	**(95% CI)**		**GM**	**(95% CI)**		**GM**	**(95% CI)**	
Sex										
Male	383	3.92	(3.66, 4.19)	**	8.37	(7.70, 9.10)	*	2.84	(2.47, 3.27)	**
Female	442	3.40	(3.18, 3.64)		7.56	(7.01, 8.16)		2.21	(1.85, 2.64)	
Study area										
Urban	660	3.70	(3.46, 3.95)		8.02	(7.40, 8.68)		2.55	(2.18, 3.00)	
Rural	165	3.44	(2.97, 4.00)		7.71	(6.58, 9.03)		2.33	(1.56, 3.47)	
Costal		-	-		-	-		-	-	
Air monitoring area		-	-		-	-		-	-	
Body Mass Index										
Underweight	109	3.81	(3.48, 4.16)		3.81	(3.48, 4.16)		3.03	(2.33, 3.93)	
Normal	544	3.60	(3.38, 3.84)		3.60	(3.38, 3.84)		2.43	(2.11, 2.79)	
Obese	172	3.73	(3.36, 4.14)		3.73	(3.36, 4.14)		2.50	(2.07, 3.02)	
Monthly household income (US $)										
Do not know	47	3.79	(3.33, 4.32)		6.82	(5.81, 8.01)		2.42	(1.97, 2.97)	
<2350	54	3.63	(3.04, 4.34)		7.64	(6.42, 9.09)		2.18	(1.50, 3.17)	
2350 to 5875	377	3.59	(3.32, 3.87)		8.17	(7.42, 8.99)		2.58	(2.21, 3.01)	
5875 to 8225	200	3.72	(3.45, 4.02)		8.00	(7.27, 8.80)		2.62	(2.22, 3.09)	
>8225	147	3.71	(3.40, 4.04)		7.97	(7.10, 8.93)		2.40	(1.99, 2.88)	
Smoking habits										
Never smoker	775	3.65	(3.43, 3.88)		7.94	(7.40, 8.52)		2.51	(2.17, 2.91)	
Former smoker	24	3.89	(3.15, 4.80)		8.29	(6.82, 10.1)		3.16	(2.26, 4.42)	
Current smoker	26	3.73	(3.16, 4.39)		8.47	(6.57, 10.9)		2.20	(1.63, 2.96)	
Drinking water										
Tap water	173	3.96	(3.54, 4.43)		7.84	(7.12, 8.64)		2.72	(2.16, 3.43)	
Use a water purifier	497	3.58	(3.37, 3.81)		8.01	(7.40, 8.67)		2.44	(2.12, 2.81)	
Bottled water	144	3.53	(3.25, 3.84)		7.99	(7.12, 8.97)		2.60	(2.06, 3.28)	
Mineral spring water	11	4.11	(3.05, 5.54)		7.89	(6.76, 9.21)		1.97	(1.45, 2.67)	
Use of nonstick kitchen tools										
No use	17	3.65	(3.00, 4.43)	*	6.93	(5.66, 8.48)		2.53	(1.87, 3.42)	*
Less than once a week	35	3.13	(2.83, 3.46)		6.52	(5.75, 7.38)		2.11	(1.41, 3.16)	
Once a week and more	480	3.63	(3.41, 3.86)		7.96	(7.37, 8.61)		2.47	(2.14, 2.86)	
Once a day and more	293	3.78	(3.50, 4.08)		8.25	(7.59, 8.96)		2.65	(2.21, 3.17)	
Consumption of Shellfish										
Not eating	279	3.38	(3.15, 3.63)	*	7.85	(7.19, 8.56)	*	2.60	(2.24, 3.01)	*
Once a month and more	434	3.70	(3.44, 3.98)		8.00	(7.31, 8.76)		2.41	(1.99, 2.91)	
Once a week and more	112	4.27	(3.90, 4.68)		8.12	(7.38, 8.92)		2.75	(2.31, 3.28)	

NOTE: Missing ≥ 19 years (*n* = 1246), 12–18 years (*n* = 3), * *p* < 0.05, ** *p* < 0.001.

## Data Availability

We used data from the Korean National Environmental Health Survey (KoNEHS) cycle 4, which was conducted by the National Institute of Environmental Research under the Ministry of Environment, Republic of Korea. The data presented are not publicly accessible. Requests for these data can be made to the National Institute of Environmental Research (knehs@korea.kr).

## Appendix A

**Table A1:** Chemical groups selected for biomonitoring in KoNEHS.

Environmental Chemical	KoNEHS 1	KoNEHS 2	KoNEHS 3	KoNEHS 4	KoNEHS 5
Metals					
Lead Mercury Cadmium	○	○	○	○	○
Manganese Molybden Nickel Antimony Vanadium Chromuim	-	-	-	-	○
Volatile Organic Compound Metabolites					
t,t-Muconic acid	○	○	○	○	○
Benzylmercapturic acid	-	-	○	○	○
Hippuric acid	○	○	-	-	-
Methylhippuric acid	○	○	-	-	-
Mandelic acid	○	○	-	-	-
Phenylglyoxylic acid	○	○	-	-	-
Polycyclic Aromatic Hydrocarbon Metabolites					
1-Hydroxypyrene	○	○	○	○	○
2-Hydroxynaphthalene	○	○	○	○	○
1-Hydroxyphenanthrene	-	○	○	○	○
2-Hydroxyfluorene	-	○	○	○	○
Phthalate and Phthalate Alternatives Metabolites					
Mono-(2-ethyl-5-hydroxyhexyl) phthalate (MEHHP)	○	○	○	○	○
Mono-(2-ethyl-5-oxohexyl) phthalate (MEOHP)	○	○	○	○	○
Mono-(2-ethyl-5-carboxypentyl) phthalate (MECPP)	-	○	○	○	○
Mono-benzyl phthalate (MBzP)	-	○	○	○	○
Mono-(carboxyoctyl) phthalate (MCOP)	-	-	○	-	-
Mono-n-butyl phthalate (MnBP)	○	○	○	○	○
Mono-(3-carboxypropyl) phthalate (MCPP)	-	-	○	○	○
Mono-(carboxynonyl) phthalate (MCNP)	-	-	○	-	-
Mono methyl phthalate (MMP)	-	-	-	○	○
Mono ethyl phthalate (MEP)	-	-	-	○	○
Environmental phenols					
Bisphenol A	○	○	○	○	○
Bisphenol F	-	-	○	○	○
Bisphenol S	-	-	○	○	○
Triclosan	-	○	○	○	○
Methyl paraben	-	-	○	○	○
Ethyl paraben	-	-	○	○	○
n-Propyl paraben	-	-	○	○	○
Butyl paraben	-	-	-	○	○
Benzophenone-3	-	-	-	○	○
Pyrethroid Insecticide Metabolites					
3-Phenoxybenzoic acid	○	○	○	○	○
Nicotine Metabolite					
Cotinine	○	○	○	○	○
Perfluoroalkyl and Polyfluoroalkyl Substances					
PFOA, PFOS, PFHxS, PFDeA, PFNA	-	-	-	○	○
Polychlorinated biphenyls, PCBs					
52, 56, 74, 101, 105, 118, 123, 126, 138, 153, 157, 167, 180, 187	-	-	-	-	○
Organochlorine pesticide, OCPs					
*p,p’*-DDT, DDE, Hexachlorobenzene, β-HCH, γ-HCH	-	-	-	-	○
Polybrominated Diphenyl Ethers, PBDEs					
28, 47, 99, 100, 153, 154	-	-	-	-	○

- Not measured.

**Table A2:** The analysis methods for environmental chemiclas in the KoNHES 4.

Category	Chemicals	Analytical Technique
Metals		
	Lead	GF-AAS (AA-800, PerkinElmer)
	Mercury	DMA (DMA-80, Milestone)
	Cadmium	GF-AAS (PinAAcle 900z, PerkinElmer)
Volatile Organic Compound Metabolites		
	t,t-Muconic acid	HPLC-MS/MS (API Triple Quad 5500, Sciex)
	Benzylmercapturic acid	
Polycyclic Aromatic Hydrocarbon Metabolites		
	1-Hydroxypyrene	GC-MS (7890A/5975C, Agilent)
	2-Hydroxynaphthalene	
	1-Hydroxyphenanthrene	
	2-Hydroxyfluorene	
Phthalate and Phthalate Alternatives Metabolites		
	Mono-(2-ethyl-5-hydroxyhexyl) phthalate (MEHHP)	HPLC-MS/MS (Agilent 6490)
	Mono-(2-ethyl-5-oxohexyl) phthalate (MEOHP)	
	Mono-(2-ethyl-5-carboxypentyl) phthalate (MECPP)	
	Mono-benzyl phthalate (MBzP)	
	Mono-carboxyoctyl phthalate (MCOP)	
	Mono-n-butyl phthalate (MnBP)	
	Mono-(3-carboxylpropyl) phthalate (MCPP)	
	Mono-carboxynonyl phthalate (MCNP)	
	Mono methyl phthalate (MMP)	
	Mono ethyl phthalate (MEP)	
Environmental phenols		
	Bisphenol A	HPLC-MS/MS (API Triple Quad 5500, AB Sciex)
	Bisphenol F	
	Bisphenol S	
	Triclosan	
	Ethyl paraben	
	Methyl paraben	
	Propyl paraben	
	Butyl paraben	
	Benzophenone-3	
Pyrethroid Insecticide Metabolites		
	3-Phenoxybenzoic acid	GC-MS (Clarus 600T, Perkin Elmer)
Nicotine Metabolite		
	Cotinine	GC-MS (Clarus 600T, Perkin Elmer)
Polyfluoroalkyl substances		
	Perfluorooctanoic acid (PFOA)	HPLC-MS/MS (Q-Sight Triple Quad, Perkin-Elmer)
	Perfluoro octane sulfonic acid (PFOS)	
	Perfluoro hexane sulfonic acid (PFHxS)	
	Perfluoro nonanoic acid (PFNA)	
	Perfluoro decanoic acid (PFDeA)	
Spot urine adjusted		
	Creatinine	Rate-blanked Jaffé kinetic assay (ADVIA 1800 Auto Analyzer, Siemens)
